# Retinoic acid is a key regulatory switch determining the difference between lung and thyroid fates in *Xenopus laevis*

**DOI:** 10.1186/1471-213X-11-75

**Published:** 2011-12-20

**Authors:** Jean H Wang, Steven J Deimling, Nicole E D'Alessandro, Lin Zhao, Fred Possmayer, Thomas A Drysdale

**Affiliations:** 1 Children's Health Research Institute, London, Ontario, Canada; 2 Department of Biology, University of Western Ontario, London, Ontario Canada; 3 Department of Obstetrics and Gynecology, University of Western Ontario, Ontario, Canada; 4 Department of Biochemistry, The University of Western Ontario, London, Ontario, N6A 5C1, Canada; 5 Department of Paediatrics, Department of Physiology and Pharmacology, The University of Western Ontario, London, Ontario, Canada

## Abstract

**Background:**

The lung and thyroid are derived from the anterior endoderm. Retinoic acid and Fgf signalling are known to be essential for development of the lung in mouse but little is known on how the lung and thyroid are specified in *Xenopus*.

**Results:**

If either retinoic acid or Fgf signalling is inhibited, there is no differentiation of the lung as assayed by expression of *sftpb*. There is no change in expression of thyroid gland markers when retinoic acid signalling is blocked after gastrulation and when Fgf signalling is inhibited there is a short window of time where *pax2 *expression is inhibited but expression of other markers is unaffected. If exogenous retinoic acid is given to the embryo between embryonic stages 20 and 26, the presumptive thyroid expresses *sftpb *and *sftpc*, specific markers of lung differentiation and expression of key thyroid transcription factors is lost. When the presumptive thyroid is transplanted into the posterior embryo, it also expresses *sftpb*, although *pax2 *expression is not blocked.

**Conclusions:**

After gastrulation, retinoic acid is required for lung but not thyroid differentiation in *Xenopus *while Fgf signalling is needed for lung but only for early expression of *pax2 *in the thyroid. Exposure to retinoic acid can cause the presumptive thyroid to switch to a lung developmental program.

## Background

The differentiation of specific organs from the endoderm is directly related to their position along the anteroposterior axis of the vertebrate embryo. After initial specification of the endoderm, wnt, nodal, bone morphogenetic protein and fibroblast growth factor (Fgf) signals quickly subdivide the gut into the foregut, midgut and hindgut [1]. Expression of *hhex, sox2 *and *foxa2 *are associated with the foregut [2] whereas expression of *sox17 *[3] and *cdx *[4] genes is eventually restricted to posterior endoderm. The foregut will give rise to the thyroid, thymus, parathyroid, lung, liver, pancreas, and stomach.

Although specific transcription factors are associated with each of these organ systems in later development, expression of key transcription factors at early stages is not restricted to a specific organ system. In the foregut, *nkx2.1 *(or *ttf1*) is required for development of both the lung and thyroid suggesting a close relationship between these tissues but each organ has a distinct set of transcription factors that are required for differentiation. The thyroid also expresses *hhex, pax8*, and *foxe1 *[5], although in *Xenopus *and zebrafish, *pax2 *is the Pax gene expressed in the thyroid [6,7]. In addition to *nkx2.1*, the lung expresses *foxa2 *and *foxp2 *[8-10]. The interplay of many different signalling pathways is necessary for defining both of these organs. Fibroblast growth factor (Fgf) signalling has been shown to be essential for both thyroid [11] and lung although the signal amplitude and timing are important for determining the output from the Fgf signal [12].

Recently, in addition to Fgf signalling, it has been demonstrated that graded levels of retinoic acid (RA) are important in chick for defining the different regions along the anterior-posterior axis of the developing endoderm [13]. The most anterior endoderm, that gives rise to the thyroid, forms with little or no RA although RA is necessary for other anterior endoderm derivatives including the lung [14,15] and pancreas [16,17]. In frog, *aldh1a2 *(*raldh2*), encoding the enzyme primarily responsible for the synthesis of RA, is expressed dynamically throughout development. After gastrulation when patterning of the gut is being established, expression is found in the lateral plate mesoderm overlying the anterior gut with highest levels on the dorsal side. There are discrete regions of expression in the head but on the ventral side, there is little expression anterior to the forming heart [18,19]. This would predict that the forming lung should be exposed to RA but that more posterior endoderm would be exposed to lower levels of RA. The most anterior endoderm, giving rise to the thyroid, should also have limited exposure to RA. Later in development, expression of *aldh1a2 *is found in specific gut regions where it is required for the gut looping process [20].

In *Xenopus*, differentiation markers that can be used to study development of the lung have only recently been identified [21]. In that study, *surfactant protein B *(*sftpb*) and *surfactant protein C *(*sftpc*) were shown to have high sequence conservation with other homologues and were surprisingly early markers of lung in *Xenopus*, being expressed as early as stage 39. *Sftpb *and *sftpc *encode important constituents of pulmonary surfactant, a material that regulates surface tension in the lung alveoli and are expressed solely in the lung [22]. Although the role of thyroid hormone in *Xenopus *biology has been extensively studied [23], the development of the thyroid is not well characterized. Expression studies have demonstrated key early transcription factors, identified as essential in other organisms, likely play a role in thyroid development in *Xenopus *including *nkx2.1 *[24], *pax *genes [6,25], *foxe1*, and *foxe4 *[25], but how the thyroid is specified in frog is not known.

The goal of this study was to test the hypothesis that the underlying mechanism for the development of the lung and thyroid in *Xenopus *is similar to other model organisms. In doing so, we demonstrate that the requirement for RA and Fgf signalling in lung differentiation is conserved in *Xenopus*. Furthermore, if exogenous retinoic acid is applied to the embryo after gastrulation, the thyroid primordium will express *sftpb *and *sftpc*. In addition, expression of early transcription factors that are normally expressed in the thyroid but not the lung, *pax2, foxe4*, and *hhex *are lost in presumptive thyroid in response to retinoic acid. These results further demonstrate that spatial control of specific signalling factors is essential for the patterning of the foregut endoderm and that retinoic acid signalling is a key element of that process.

## Results

### Temporal expression of lung and thyroid markers

**Figure 1:**
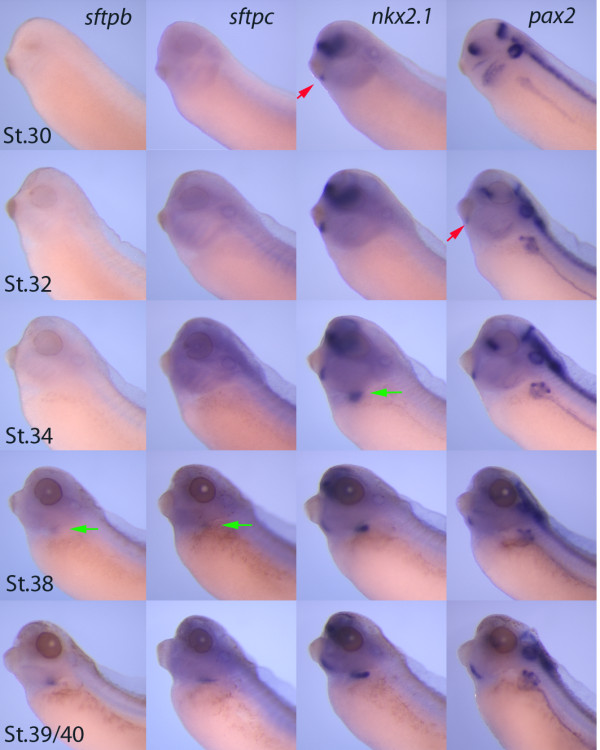
**Temporal expression of lung and thyroid markers**. Whole mount *in situ *hybridization was used to compare the temporal expression of key early genes in the development of the thyroid and lung. Expression of *nkx2.1 *is first detectable in the thyroid (red arrows) at stage 30 and is subsequently expressed in the lung (green arrows) at stage 34. Expression of *pax2 *begins at stage 32 in the thyroid. Expression of the differentiation markers *sftpb *and *sftpc *is first detectable at stage 38.

To establish a baseline of when the different markers of lung and thyroid are first detectable, their expression was compared by whole mount *in situ *hybridization (Figure 1). *Nkx2.1 *expression was first detectable in the thyroid at stage 30 and appeared as a small dot immediately caudal to the cement gland. There was only a slight delay before expression of *pax2 *was detected in the thyroid. At stage 34, expression of *nkx2.1 *was detectable in the forming lung. As expected, the expression of these key transcription factors preceded the expression of the lung differentiation markers *sftpb *and *sftpc *both of which were detectable at stage 38.

### Retinoic acid and FGF are required for lung development in *Xenopus*

**Figure 2:**
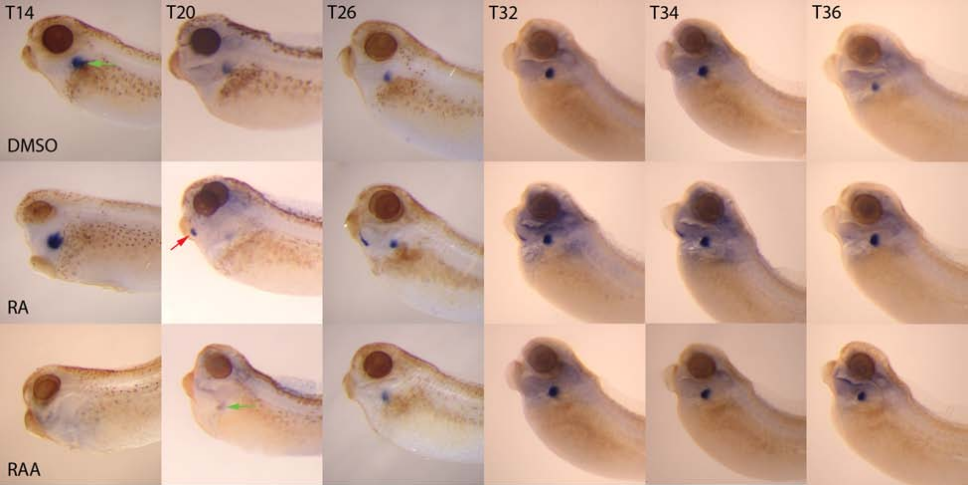
**Retinoic acid is required for lung differentiation but excess retinoic acid causes expression of lung differentiation markers in the thyroid**. When embryos were treated with a retinoic acid antagonist (RAA) at stage 14 of development, the lung failed to differentiate as judged by the lack of *sftpb *expression at stage 38/40. If the retinoic acid antagonist is not added until stage 26, the expression of *sftpb *in the lung was essentially normal. If embryos were exposed to exogenous retinoic acid (RA) starting between stages 20 to 34, expression of *sftpb *was detectable in both the lung (green arrows) and in the thyroid (red arrow). Addition of retinoic acid at earlier (stage 14) or later stages (stage 36) did not result in expression of *sftpb *in the presumptive thyroid. Carrier control (DMSO) embryos had normal expression of *sftpb*. Treatment times are indicated at the top of each column (eg. T-st14 indicates that the treatment was initiated at embryonic stage 14).

A requirement for both RA and Fgf signalling has been demonstrated in mouse lung development [26-29] and we wished to confirm that this requirement is conserved in *Xenopus*. Using a pan-retinoic acid receptor antagonist [30], we were able to show that there is a requirement for RA signalling. However, the ability of the antagonist to suppress lung differentiation, as assayed by *sftpb *expression is restricted to very early stages of lung development. If RA signalling was blocked at stage 26, well before the expression of *nkx2.1*, lung differentiation occurred normally (Figure 2). Expression of *sftpb *was detectable in some embryos when RA signalling was blocked as early as stage 20, although most had no expression and those that did had lower expression levels than control embryos. These results are mirrored by the expression of *nkx2.1*. If RA signalling is blocked prior to stage 20, *nkx2.1 *expression is lost in the lung although maintained in the thyroid. At stage 20 there is lung expression in some embryos and if treatment with the RA antagonist is delayed until stage 26, essentially all embryos express *nkx2.1 *in the lung.

**Figure 3:**
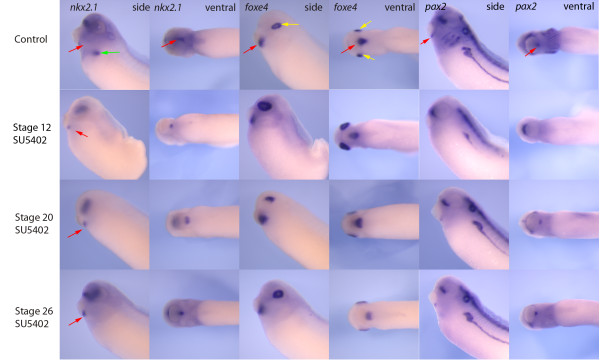
**Fgf signalling is required for lung and elements of thyroid differentiation in *Xenopus***. When embryos were treated with SU5402 at either stage 12, 20 or 26, expression of nkx2.1 was lost in the lung (green arrows) but not the thyroid (red arrows) or anterior neural tissue. Although treatment with SU5402 at stage 20 caused clear deformations in the lens ectoderm expression (yellow arrows) of *foxe4*, there was no clear effect on the thyroid expression of *foxe4*. Expression of *pax2 *in the thyroid was lost when SU5402 was applied at stage 12 but expression of *pax2 *was observed in most embryos at stage 20 and all embryos when treated at stage 26. Note the severe tail defects in embryos treated with SU5402 at stage 12 demonstrating the effectiveness of the block to Fgf signalling. (side - side view, ventral - ventral view).

If Fgf signalling was blocked using the inhibitor SU5402 after stage 12, 20, or even stage 26, expression of *nkx2.1 *was lost in the lung region whereas the expression in the forming thyroid was maintained (Figure 3). Again, as expected, blocking Fgf signalling also resulted in a loss of *sftpb *expression, although some embryos did show some very weak *sftpb *expression (Additional File 1, Figure S1). The loss of Fgf signalling was also confirmed by looking at the expression of *sprouty2*, a direct target of Fgf signalling (Additional File 1, Figure S1). In addition, expression of *foxe4 *was maintained in the forming thyroid in the presence of SU5402. This suggests that Fgf signalling was required for lung specification but not thyroid specification at the times tested. Expression of *foxe4 *in the forming lens was maintained when FGF signalling was blocked but the lens morphology was aberrant when compared to controls in Fgf inhibitor treatments that began at stage 20 (Figure 3). Given that Fgf signalling has been implicated in thyroid development in other organisms [7], we also looked at the expression of the other early thyroid marker, *pax2*. We found that blocking Fgf signalling immediately after gastrulation (stage12) resulted in no thyroid expression of *pax2*, although expression in other tissues remained unaffected (Figure 3). When Fgf signalling was blocked at stage 20, *pax2 *expression in the thyroid was apparently normal, although expression was not observed in all embryos. Treatment at stage 26 had no effect on *pax2 *expression in the thyroid.

### Exogenous retinoic acid causes the thyroid to express the lung developmental program

As the requirement for RA was very early in the process of lung organogenesis, one potential role for retinoic acid could be to determine the number of lung progenitors. Therefore, we also treated embryos with exogenous retinoic acid to see if there was an increase in the size of the developing lung. Although we did not see any clear increase in lung size, there was ectopic expression of *sftpb *in a small region of the embryo that corresponded both in size and shape with the developing thyroid (Figure 2). Addition of RA was able to cause this ectopic expression even when retinoic acid was added as late as stage 34 and the activity was dose dependent (Table 1). Concentrations of RA that were able to cause ectopic expression of *sftpb *were the same as those that caused loss of *pax2 *expression in the thyroid suggesting that the two processes might be linked (Table 1). When embryos were treated with RA at stage 14, there was no ectopic expression of *sftpb *posterior to the cement gland (Figure 2) although there was also no expression of *pax2 *or *foxe4*.

**Table 1:** Presence of *pax2 *and *sftpb *expression in the presumptive thyroid exposed to different concentrations of RA

	1 μM RA	0.1 μM RA	0.01 μM RA	DMSO control
*pax2*	0/60	32/61	54/64	54/54
*sftpb*	74/83	63/84	37/81	0/75

The numbers represent the number of embryos expressing the gene in the thyroid over the total number of embryos examined.

**Figure 4:**
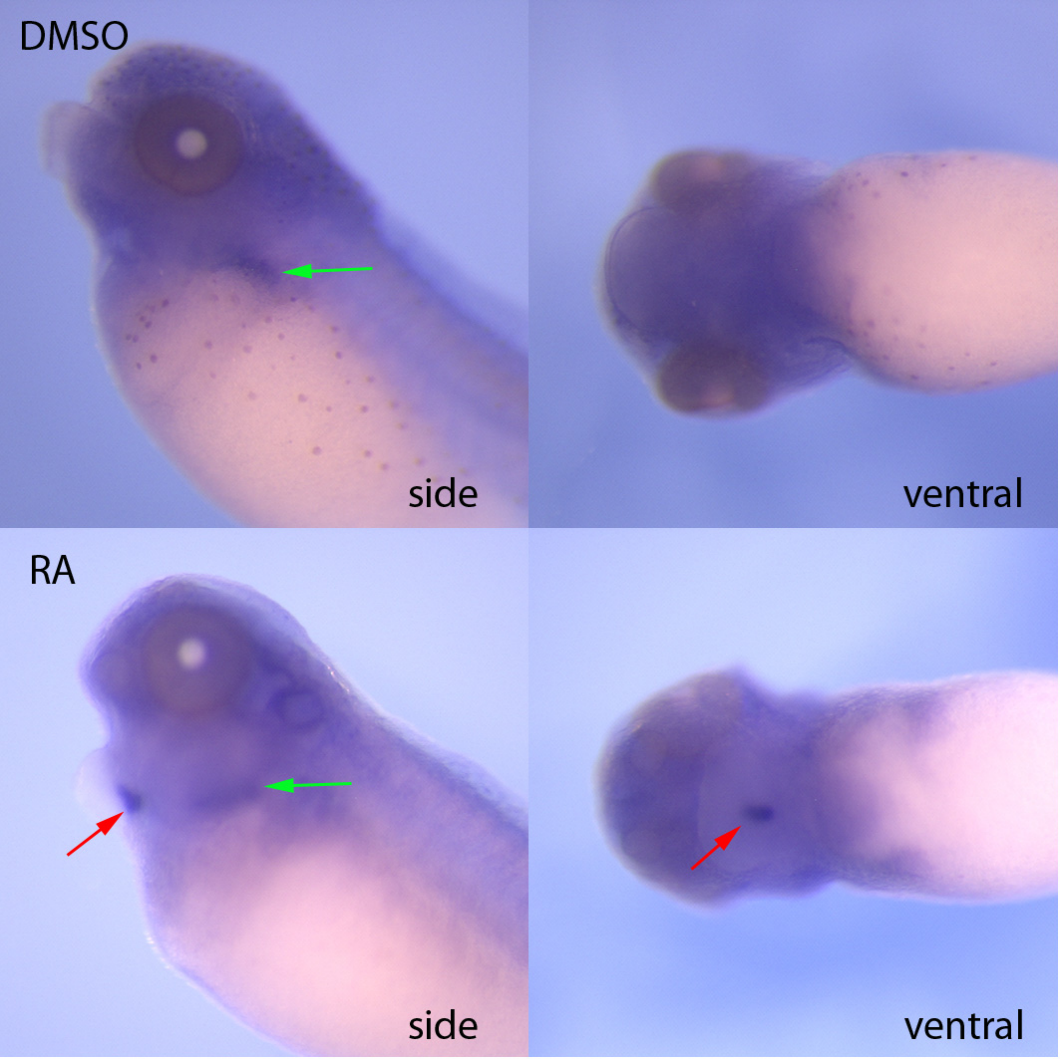
**Exogenous retinoic acid causes ectopic expression of *sftpc *in the presumptive thyroid**. Whole mount *in situ *hybridization for *sftpc *demonstrated that embryos treated at stage 26 with exogenous retinoic acid showed ectopic expression of *sftpc *in the developing thyroid (red arrows) as well as the normal expression in the lung (green arrows). (side - side view, ventral - ventral view).

**Figure 5:**
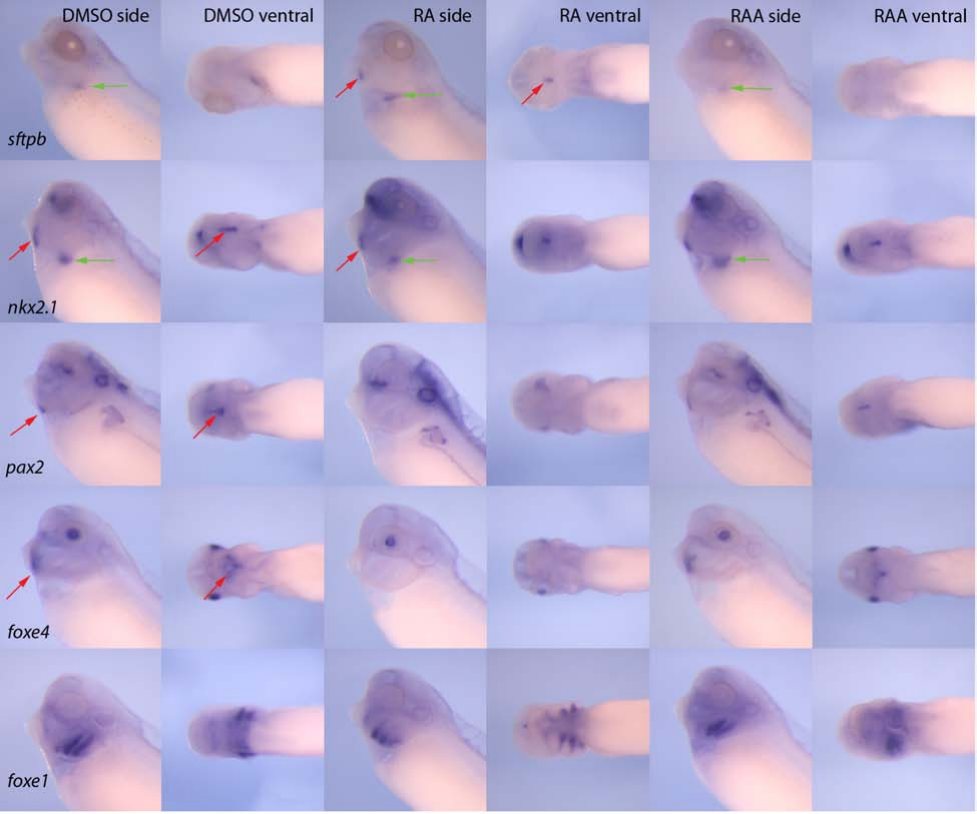
**Exogenous retinoic acid signalling results in the loss of early thryroid gene expression**. When embryos are treated with retinoic acid (RA) at stage 24/26, expression of *sftpb *was detected in the thyroid (red arrows) and in the lung (green arrows) but it is only expressed in the lung in control (DMSO) embryos. *Pax2 *and *foxe4 *are normally expressed in the thyroid although both have distinct expression domains in other tissues. Embryos treated with RA still expressed both *pax2 *and *foxe4 *but the expression in the thyroid was eliminated. Expression of *foxe1 *could be observed in all embryos, particularly in the pharynx, but expression in the thyroid was undetectable. Treatment with the retinoic acid antagonist at stage 26 did not eliminate the expression of any tested markers in either the thyroid or lung.

In order to determine if this simply represented ectopic expression of *sftpb *in the thyroid or a more global change towards lung differentiation in the thyroid, we examined expression of *sftpc*, another highly specific lung specific marker and found that it was also expressed in the thyroid primordium when embryos were exposed to RA (Figure 4). The presence of *sftpb *and *sftpc *mRNA suggests either that RA causes lung differentiation markers to be expressed in the thyroid or that it causes the conversion of presumptive thyroid tissue into lung. To distinguish between these possibilities, we examined the expression of the key early transcription factors in lung and thyroid development in response to treatment with retinoic acid. As *nkx2.1 *is expressed in both the early thyroid and lung, it was perhaps not surprising that there was no difference in expression of *nkx2.1 *when embryos were exposed to RA or when RA signalling was blocked using the RA inhibitor at stage 26 (Figure 5). However, *pax2 *and *foxe4*, both markers of the early thyroid are not expressed in the thyroid in RA treated embryos, although expression at other sites is not lost in the same embryos. *Foxe1 *has been described as another early marker of the thyroid [25] but we find that it does not have the same strong midline expression seen with other thyroid markers, although we cannot rule out low levels of expression. Nevertheless, RA had no discernable effect on expression of *foxe1 *in that region although some patterning differences were noted in the pharyngeal region (Figure 5).

**Figure 6:**
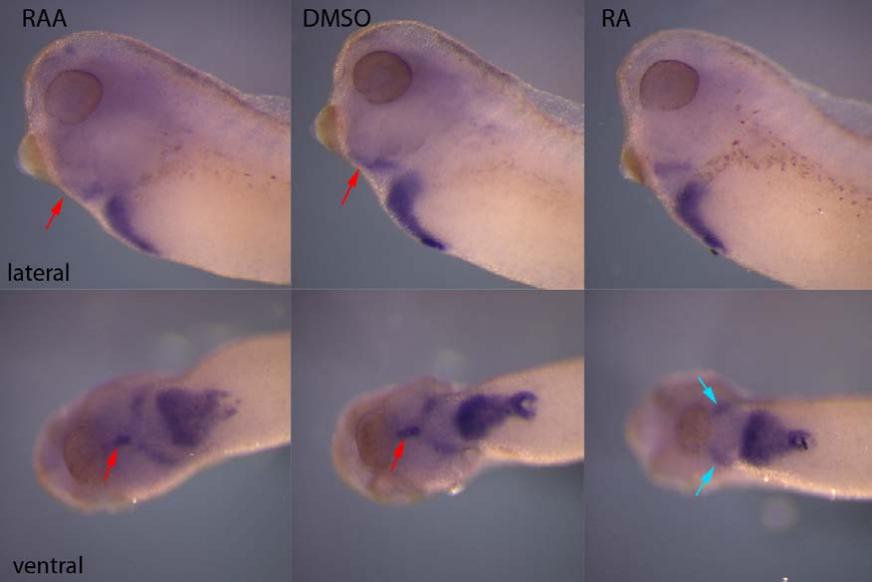
**Treatment with retinoic acid eliminated expression of *hhex *in the thyroid but not liver**. When embryos were treated at stage 24/26 and cultured to stage 36, expression could be detected near the thyroid even in RA treated embryos but in ventral view it can be clearly seen that midline expression of *hhex *(red arrow) is eliminated by RA but there is an additional region of expression lateral to the thyroid (blue arrows) that is not lost when embryos were treated with RA. There is also no obvious change in the expression of the large liver expression domain found caudal to the thyroid expression.

*Hhex *is a homeobox-containing transcription factor that is first expressed in a broad domain in the anterior endoderm and expression then becomes restricted to the forming thyroid and liver [1,31]. Exposure to retinoic acid during late gastrulation is known to eliminate *hhex *expression in the thyroid [16] and we wanted to ensure that our later retinoic acid treatments would still result in loss of thyroid expression. Treatment with RA at stage 26 was also able to eliminate the expression of *hhex *in the thyroid without loss of expression in the liver (Figure 6), further suggesting that RA blocks the full repertoire of early thyroid transcription factors. We also examined whether blocking RA signalling would change *hhex *expression. If RA signalling was blocked between stage 14 and 26, there was no obvious change in *hhex *expression in either the liver or thyroid (Figure 6).

Although addition of RA as late as stage 34 resulted in expression of *sftpb *in the presumptive thyroid, if the retinoic acid was added between stages 32 and 34, there was no loss of *pax2 *and *foxe4 *expression. This suggests that the tissue becomes ambiguous, expressing transcription factors that define it as thyroid in addition to the lung differentiation markers (Table 2).

**Table 2:** Effect of RA on presence of *sftpb, pax2 *and *foxe4 *expression in specific organs

	*sftpb *expression		*pax2 *expression		*foxe4 *expression	
Treatment	Lung	Thyroid	Thyroid	Kidney	Thryoid	Eye
St. 14 DMSO	33/33	0/33	10/10	10/10	11/11	11/11
St. 14 RA	32/32	0/32	0/11	11/11	0/10	0/10
St. 26 DMSO	82/82	0/82	70/70	70/70	50/50	50/50
St. 26 RA	81/82	78/82	0/74	74/74	0/54	54/54
St. 30 DMSO	22/22	0/22	12/12	12/12	9/12	12/12
St. 30 RA	24/26	20/26	2/11	11/11	2/11	11/11
St. 32 DMSO	42/42	0/42	26/26	26/26	24/24	24/24
St. 32 RA	39/40	39/40	21/25	25/25	23/23	23/23
St. 34 DMSO	6/6	0/6	10/10	10/10	28/28	28/28
St. 34 RA	5/5	5/5	10/10	10/10	16/16	16/16
St. 36 DMSO	6/6	0/6	10/10	10/10	10/10	10/10
St. 36 RA	6/6	0/6	10/10	10/10	10/10	10/10

The numbers represent the number of embryos expressing the gene in the organ examined over the total number of embryos examined.

**Figure 7:**
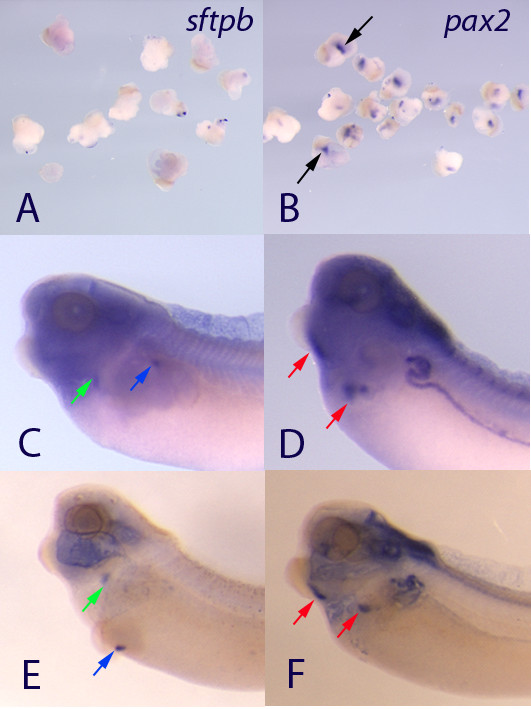
**Transplantation of the presumptive thyroid explants into the embryo flank is sufficient to cause expression of *sftpb *in the explant**. When explants of the presumptive thyroid region are cultured alone, they do not express *sftpb *(A) but do express *pax2 *(B - black arrows). Note that the small blue flecks are seen on the surface of many embryos due to the stickiness of the cement gland that is also usually in part of the explant. When those same explants are placed on to the flank region immediately after they are removed from donor embryos, *sftpb *expression can be seen in the explant (blue arrows) as well as the endogenous lung (green arrows). When explants are transplanted, expression of *pax2 *(red arrows) can be seen in the explants. Embryos in C and D are normal views and embryos in E and F have been cleared to better visualize staining although cavity staining is also seen when embryos are cleared.

One simple model for explaining differentiation of the thyroid might be that gastrulation movements position the presumptive thyroid far enough towards the rostral end of the embryo that it is not exposed to retinoic acid. If so, we would predict that simply transplanting the presumptive thyroid to the flank of the embryo, where there is robust *aldh1a2 *expression, may be sufficient to cause those precursors to differentiate as lung. This would also directly test whether endogenous levels of retinoic acid would be sufficient to cause the thyroid to lung switch. When presumptive thyroid explants were removed and cultured to stage 38 they did not express *sftpb *but robust expression of *pax2 *could be seen in the cultured explants (Figure 7A,B). When explants were transplanted into the flank of a donor embryo and allowed to heal, the explants could be seen to express *sftpb *(Figure 7C, E) suggesting that endogenous levels of RA can cause the presumptive thyroid to express *sftpb*. However, the transplants also continued to express *pax2 *suggesting that, similar to when RA is added between stages 30 and 34, the tissue has not lost all thyroid characteristics (Figure 7D,F). The size of the expression domain also corresponded well with the normal size of the thyroid suggesting that it was only the presumptive thyroid domain that was expressing *sftpb*.

## Discussion

### RA and FGF are required for lung development in *Xenopus*

Little is known about the signalling mechanisms required for lung development in *Xenopus*. A recent study has suggested that wnt and hedgehog signalling are needed for later lung development based on the expression patterns of *shh, wnt7b, wnt5a *and *wif1 *but their functional roles have yet to be elucidated. Here we show that both RA (Figure 2) and Fgf (Figure 3) signalling are necessary for differentiation of the lung in *Xenopus*. Both of these signalling systems are required for lung differentiation in the mouse and the two systems have been linked as loss of RA results in reduced levels of Fgf10 and subsequent lung agenesis [14]. It would appear that both RA and Fgf are very high in the hierarchy of lung specification as both are required well before the initial expression of *nkx2.1*.

The requirement for RA signalling is lost quite early in the development of the lung. If the antagonist is added as early as stage 20, well before the expression of *nkx2.1*, there is still detectable expression of *sftpb*. If the antagonist is added at stage 26, the expression of *sftpb *is essentially the same as control embryos. The *sftpb *promoter in humans has binding sites for both retinoic acid receptors and for nkx2.1 and the two transcription factors act synergistically to drive expression of *sftpb *[32]. Although we cannot make any conclusions regarding the levels of *sftpb *expression using whole mount *in situ *hybridization, our results demonstrate that the activation of the retinoic acid receptors by RA is not a requirement for *sftpb *expression.

The requirement for Fgf also occurs early in *Xenopus *as blocking Fgf signalling at stage 20 causes a loss of *nkx2.1 *expression, although unlike RA, the requirement is maintained later as blocking at stage 26 still showed a complete loss of *nkx2.1 *(Figure 3) and subsequent *sftpb *expression (Additional File 1, Figure S1) in the lung.

### Requirements of RA and Fgf signalling for development of the thyroid gland

When RA signalling was blocked, expression of thyroid markers was not affected (Figure 5). In mouse, Fgf8, regulated by tbx1 and arising from the secondary heart field, is required for normal thyroid development although loss of fgf8 did not eliminate the thyroid [11]. In zebrafish, hand2 is upstream of *fgf8 *and this pathway is essential for thyroid development and again it is the cardiac mesoderm that is the source of the Fgf signal [7]. Thus it is surprising that the SU5402 treatments had little effect on expression of either *nkx2.1 *or *foxe4*, although it was clear that SU5402 was able to cause developmental defects in the treated embryo, including loss of *nkx2.1 *expression in the lung, severe loss of tail (Figure 3) and loss of *sprouty2 *expression (Additional File 1, Figure S1).

We did find that the expression of *pax2 *in the thyroid was lost with early SU5402 treatments. The thyroid itself does not appear to be a direct target of the Fgf signal in zebrafish [7]. If the potential intermediate tissue were already established before we treated with SU5402 in *Xenopus*, this could provide an explanation for the lack of effect on *nkx2.1 *and *foxe4 *expression. Identification of the intermediate tissue is needed in order to test this possibility. The lack of *pax2 *expression while *nkx2.1 *and *foxe4 *expression is maintained is perhaps expected. In *pax8 *knockout mice, the early thyroid primordium is maintained with normal *nkx2.1 *expression although the thyroid is eventually lost at later stages due to apoptosis [5,33]. Thus, eventual loss of the thyroid might eventually be expected in *Xenopus *when Fgf signalling is blocked at very early stages.

### RA causes presumptive thyroid tissue to express lung-specific markers

We have observed that addition of exogenous RA results in expression of *sftpb *and *sftpc *in the thyroid (Figure 2,4), both of which are clear markers of the differentiated lung. This does not seem to be simple ectopic expression as thyroid expression of *hhex, pax2*, and *foxe4 *are lost with the addition of RA (Figure 5,6). *Hhex, pax8, foxe4*, and *nkx2.1 *have been identified as forming a key transcription factor network for maintaining the thyroid [5] and it appears that a similar network exists in *Xenopus. Pax2 *substitutes for the expression of *pax8 *[6] and based on our expression data, it appears that *foxe4 *is more closely related to the central thyroid region than *foxe1 *(Figure 5). Nevertheless, the early thyroid markers that we have examined are lost with the addition of RA suggesting that there has been a loss of the thyroid developmental program. The one exception is that expression of *nkx2.1 *is maintained but it is also normally expressed in the lung. The suppression of *hhex *expression by exogenous RA has also been observed in the chick although in that system there was also loss of *nkx2.1 *expression [13] suggesting that RA is sufficient to block thyroid development but not able to cause the fate switch that we observe in *Xenopus*. In the same chick study it was concluded that the thyroid must form in the absence or at very low levels of RA [13]. Our results confirm this and suggest that in *Xenopus*, the absence of RA is actually required for thyroid development, as presence of RA would result in potential differentiation as lung tissue.

We were not able to look at any differentiation markers of thyroid. Although thyroid hormone has been extensively studied in terms of metamorphosis in *Xenopus*, little is known about the ontogeny of the enzymes required for thyroid hormone production. A study examining the expression of thyroid peroxidase, type II idothyronine deiodinase, and type III iodothyronine deiodinase showed that none of these were expressed in the thyroid before stage 41 [34]. Interestingly, although initially described as thyroid gland expression, thyroid peroxidase is expressed in the lung at stage 43 [34] further demonstrating the close relationship between these two organs. We also hoped to determine whether RA-treated thyroid progenitors would form structures reminiscent of a mature lung such as branching or alveoli. However, these are very late events in *Xenopus *development [35] and embryos treated with RA usually developed swelling around the heart that did not allow culturing to later stages.

The recent finding that sfrp5 and wnt11, both linked to the planar cell polarity pathway [36,37], are necessary for foregut specification suggests that morphogenetic movements of the endoderm may be important for proper specification of the different organ systems [38]. The requirement for morphogenesis suggests a relatively simple model whereby the early movements of the endoderm simply result in the furthest anterior endoderm, the presumptive thyroid, being beyond the mesodermal domains where retinoic acid is being synthesized. By simply physically moving the presumptive thyroid endoderm back to regions that would be predicted to have high levels of RA, the transplanted endoderm was able to express lung differentiation markers (Figure 7). However, we cannot rule out the possibility that other factors, present in the posterior embryo, might also be responsible for the transition.

## Conclusions

Both RA and FGF signalling are required for the differentiation of the lung in *Xenopus *embryos. RA is required for lung development before the lung primordium expresses *nkx2.1 *and *sftpb *can still be expressed in embryos that are treated with an RA antagonist in later development. If the presumptive thyroid is exposed to RA before stage 20, early thyroid transcription factors are suppressed and lung differentiation markers will be expressed in the presumptive thyroid.

## Methods

### Embryo Collection

Female *Xenopus laevis *frogs were injected with 600-700 IU of human chorionic gonadotrophin (Sigma) to induce ovulation. *In vitro *fertilization of ovulated eggs was performed in 80% Steinberg's solution containing minced testis. Embryos were dejellied with 2.5% cysteine, pH 8.0, and cultured in 20% Steinberg's solution. Embryos were staged according to the Nieuwkoop and Faber staging table [35].

### Cloning of *sftpb *and *sftpc*

Adult *Xenopus laevis *lung cDNA was used to amplify both *sftpb *and *sftpc*. The initial clones were amplified by polymerase chain reaction using the following sequences: *sftpb *forward 5'-cagtgggtccacaggatgac-3' and reverse 5'-gacggccccacactctttag-3' and *sftpc *forward 5'-ggctgcacatgagtcaaaaac-3" and reverse 5'-ctgttccggatccatttgtg-3'. The sequence obtained from the *sftpb *amplicon was used to generate new primers for a 5' rapid amplification of cDNA ends procedure that gave a longer cDNA clone that included the start codon. Sequencing of the clone showed that it matched previously identified *sftpb *sequence (Genbank Ref. NM_001096917.1). The *sftpc *sequence matched the previously identified *sftpc *sequence (Genbank Ref NM_001096721.1).

### Embryo Treatments

Embryos were treated with 1μM *all-trans *RA (Sigma) or 1μM pan RAR antagonist (Allergan #193109) [30,39] in 20% Steinberg's solution at various stages depending on the experiment. Stock solutions for both RA and RAA were 1mM dissolved in DMSO, and therefore a control treatment was performed with 0.1% DMSO in 20% Steinberg's solution. Fgf signaling was inhibited by the addition of 10μM SU5402 (EMB Biosciences) in the presence of 100 μM ATP. Control treatments were DMSO and ATP alone.

Explants were removed at stage 18/20 using the cement gland as a guide. Explants were cut and maintained in 1 × MBS. Explants were transplanted by creating a small wound in the recipient embryo at the site for transplantation and the transplantation was also conducted in 1× MBS. After the explant had healed, the embryos were transferred back to 20% Steinberg's solution.

### *In situ *Hybridization

Whole-mount *in situ *hybridizations were performed according to [40] with modifications: embryos were blocked in Tris buffered saline, 20% heat treated sheep serum; and the Proteinase K, and RNase A steps were omitted. Antisense Dig-labelled riboprobes for *sftpb *and *sftpc *were generated by cutting the plasmids with *Xba1 *and transcribing with SP6 RNA polymerase according to established protocols [40]. Antisense Dig-labelled riboprobes for *pax2a *[6], *foxe1, foxe4*, [25], *nkx2.1 *[24], and *hhex *[41] were also generated using the same methods. BM Purple (Roche Diagnostics) was used as the alkaline phosphatase substrate throughout and embryos were fixed for twenty minutes in MEMPFA before removing endogenous pigment in 1% hydrogen peroxide, 5% formamide, and 0.5%SSC for several hours after the colour reaction. For some images, the embryos were cleared by immersion in 1 part benzyl alcohol: 2 parts benzyl benzoate after dehydration in methanol. Embryos were visualized using a Leica MZ12 dissecting microscope and images were taken using Northern Eclipse software (Empix Imaging; Mississauga, ON, Canada).

## Authors' contributions

JHW carried out the majority of experiments on the embryos. SJD did the analysis of Fgf requirements. NED did the analysis of *hhex *expression. LZ cloned the *Xenopus sftpb *and *sftpc *sequences and characterized them. FP participated in the design and interpretation of experiments. TAD participated in the design and coordination of all experiments and prepared the draft manuscript. All authors read and approved the final manuscript.

## Supplementary Material

Additional file 1 **Treatment with SU5402 causes a loss of both sftpb and sprouty2 expression**. Embryos treated with SU5402, in order to block Fgf signalling, do not express *sftpb *indicating that there is a loss of differentiated lung. The location of the differentiated lung (green arrow) can be seen in the control embryo (DMSO treated). Expression of *sprouty2 *(*spry2*) was used to demonstrate the effectiveness of the Fgf signalling block. *Sprouty2 *is a target of Fgf signalling and strong expression can normally be seen at the midbrain-hindbrain border (yellow arrow) and in the pharynx (purple arrow). The expression of *sprouty2 *in these regions is effectively eliminated by addition of SU5402 at all times tested. Treatment times are indicated at the top of each column (eg. T-st12 indicates that the treatment was initiated at embryonic stage 12). Click here for file
