# Supplementary figures and images for: Retinoic acid is a key regulatory switch determining the difference between lung and thyroid fates in Xenopus laevis
Source: BMC Dev Biol. 2011 Dec 20;11:75. doi: 10.1186/1471-213X-11-75 (PMC3268113; doi:10.1186/1471-213X-11-75)

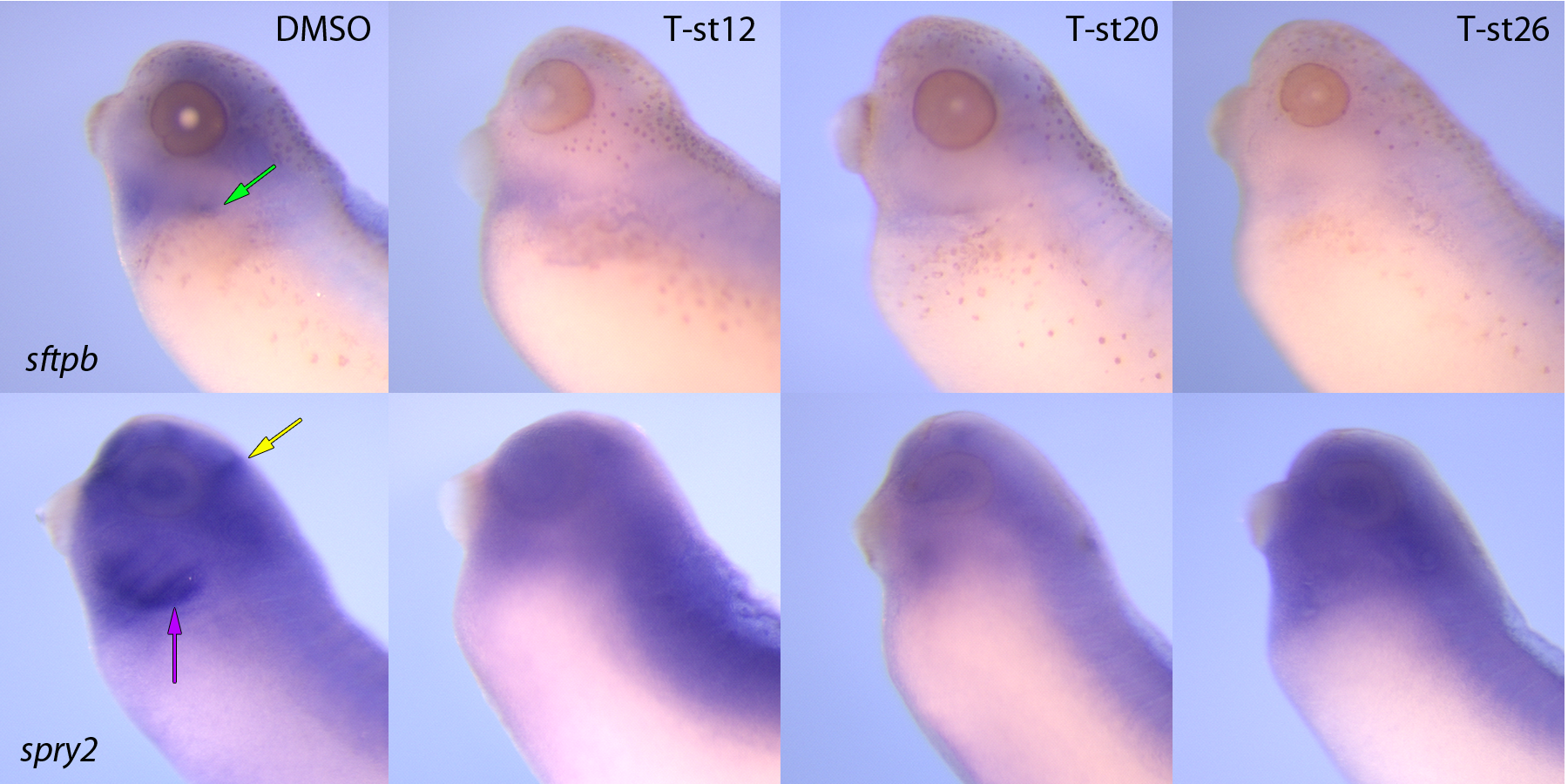

Supplement: Additional file 1 — Treatment with SU5402 causes a loss of both sftpb and sprouty2 expression. Embryos treated with SU5402, in order to block Fgf signalling, do not express sftpb indicating that there is a loss of differentiated lung. The location of the differentiated lung (green arrow) can be seen in the control embryo (DMSO treated). Expression of sprouty2 (spry2) was used to demonstrate the effectiveness of the Fgf signalling block. Sprouty2 is a target of Fgf signalling and strong expression can normally be seen at the midbrain-hindbrain border (yellow arrow) and in the pharynx (purple arrow). The expression of sprouty2 in these regions is effectively eliminated by addition of SU5402 at all times tested. Treatment times are indicated at the top of each column (eg. T-st12 indicates that the treatment was initiated at embryonic stage 12). [file 1471-213X-11-75-S1.TIFF]
